# Optimization of jasmonates bioproduction

**DOI:** 10.1186/1753-6561-8-S4-P208

**Published:** 2014-10-01

**Authors:** Alexandre Zanelli dos Santos, Murilo Daniel de Mello Innocentini, Miriam Verginia Lourenço

**Affiliations:** 1 Biotechnology Unit, UNAERP, University of Ribeirao Preto, SP, Brazil; 2 Chemical Engineering Course, UNAERP, University of Ribeirao Preto, SP, Brazil

## Introduction

Several studies have shown that jasmonates (jasmonic acid, (+)-7-isojasmonic acid and methyl jasmonate) exhibit potential anticancer activity, with the advantage of displaying selective cytotoxicity to cancer cells that spares normal lymphocytes to this function [1]. This class of compounds is present in several families of plants, being also produced by certain microorganisms, including *Gibberela fujikuroi *and *Botryosphaeria rhodina*. Bioprocess studies based on the use of strains of *B. rhodina *have shown the potential of this microorganism to produce jasmonates under controlled conditions [2]. There are several factors that affect the productivity of a fermentative process, including the strain of the microorganism, the inoculum, and the morphological and rheological properties of the broth [3]. Once selected the most producing strains, it becomes necessary to define the conditions of the fermentation process, and the standardization of the inoculum is of fundamental importance when seeking increased productivity. In view of the morphology of filamentous fungus *B. rhodina *and the difficulty to induce sporulation, the standardization of the inoculum is a major challenge. In this context, the aim of this work was to conduct the standardization of *B. rhodina *inoculum preparation for jasmonate bioproduction.

## Methodology

For the inoculum preparation, the fungus was grown in culture medium BD for 7 days, then the medium was drained and added to autoclaved distilled water. The mycelium was disintegrated and homogenized using a Turrax homogenizer (Marconi) and subsequently the DO was adjusted to 0.5 (l = 700 nm). Fermentations were conducted in 250 mL Erlenmeyer flask containing 50 mL culture medium inoculated with 5 mL of fungal homogenate M2 or 1/8 of the mycelial plate (control). Fermentations were conducted in the dark at 30°C under static conditions for 14 days. For quantification of jasmonates produced at the end of the fermentation period, the fermented broth was recovered by vacuum filtration, adjusted to pH 3.0 with 4 M HCl and then subjected to extraction with ethyl acetate. The quantification of jasmonates was performed by HPLC (High Performance Liquid Chromatography) using a chromatograph Shimadzu (LC-10AD VP) coupled to a diode array detector. A Supelcosil C_18 _column (25 cm × 4.6 mm id, 5 mm) was used and the solvent system was MeOH:Acetic Acid at 0.1% (60:40). The solvent flow was 0.85 mL min^-1 ^and the analysis monitored at 210 nm. For quantification, the external standard method was used, by plotting a calibration curve with standard AJ solutions.

## Results and conclusions

From the obtained data it was observed that the flasks inoculated with fungal homogenate had a higher concentration of jasmonic acid when compared to the control, in which 1/8 of mycelial plate was used. The average AJ concentration in the media inoculated with homogenate was 352.4 mg.L^-1^, whereas the control was 274.0 mg.L^-1^, representing a 28.6% increase in the amount of produced AJ.
